# Cortical Blindness Due to Neurocysticercosis in an Adolescent Patient

**DOI:** 10.3390/tropicalmed7060096

**Published:** 2022-06-08

**Authors:** Nnennaya U. Opara

**Affiliations:** Department of Emergency Medicine, Charleston Area Medical Center, Institute for Academic Medicine, Charleston, WV 25304, USA; nnennaya.opara@camc.org

**Keywords:** neurocysticercosis, *Taenia solium*, cortical blindness, seizures

## Abstract

Neurocysticercosis (NCC) is a common cause of recent-onset seizures in both adults and children in tropical areas, especially when there is no other suggestion of another underlying neurological disorder. In addition, there have been reports of very rare cases of bilateral cortical blindness caused by this helminth in children. It is still unclear whether healthy adolescents with no pre-existing health problems could be vulnerable to developing such sequelae due to NCC. We report a case of a 14-year-old African boy from Nigeria with bilateral cortical blindness caused by NCC due to *Taenia solium*. According to the boy’s mother, symptoms began with headaches, vomiting, fatigue, visual loss, and fever (40.0 °C). Clinical investigations led to a diagnosis of cortical blindness and encephalitis due to NCC. Appropriate treatment was administered, and it resulted in the resolution of most symptoms, though the patient remained permanently blind.

## 1. Learning Points

- NCC can produce complex neurological signs and symptoms, and, in most cases, have an indolent course.
- It is a treatable and a preventable disease with prompt intervention to prevent permanent neurological complications.
- The possible effects of this infectious disease should always be considered during the diagnostic work-up of any patient with symptoms suggestive of neurological, personality, or cognitive disorders, particularly among patients who have traveled to or lived in an endemic area.

## 2. Background

Humans contract cysticercosis through the fecal-oral route by ingesting *T. solium* eggs from tapeworm-contaminated surfaces or carriers with taeniasis [1]. When humans become infected by ingesting undercooked meat contaminated with cysticerci, the cysticerci develop into adult tapeworms in the small intestines over a period of two months. In the human gut, the adult tapeworms produce proglottids that later become gravid. The gravid proglottids eventually detach from the tapeworm and are excreted in the stool. Free eggs shed from the gravid proglottids can (in few occasion) be detected in the stool either with simple microscopy of a stool sample or with special staining [2], Since the neurological symptoms of neurocysticercosis (NCC) are nonspecific, it is difficult to diagnose the disease based on clinical findings alone [2].

NCC develops when metacestodes of *Taenia solium* spread through the bloodstream and are seeded in the brain. The presence of degenerating cysticerci causes localized edema in the affected part of the brain, which can manifest as different neurological symptoms/deficits. NCC is prevalent in Africa; it raises concerns about public health and economic livelihoods as it causes significant morbidity and mortality on the continent [3]. The climate also contributes to parasite transmission in Africa. The sub-Saharan African regions have a high level of heterogeneities in co-infection with other helminths (roundworms, hookworms) which are influenced primarily by arid temperature, elevation, and close proximity to livestock (pig farms) and bodies of water as reliable predictors of spatial distribution of tapeworms and co-infections [3,4].

## 3. Case Description

We present a case of a 14-year-old African boy from Nigeria who presented to the hospital in Ogun state, Nigeria, with symptoms of throbbing headaches, vomiting, fever (40.0 °C), slurred speech, seizures, hemiparesis, bilateral vision loss, suprapubic pain, and confusion. The symptoms had begun 3 days before the hospital visit. The patient’s parents thought the symptoms were of malaria and started him on an antimalarial drug (hydroxychloroquine) and Tylenol (acetaminophen) for the pain. However, when the patient’s symptoms failed to improve, and he complained of vision loss in both eyes. He was brought to the hospital for treatment.

Neurological examination upon arrival revealed bilateral vision loss and hemiplegia in the left part of both upper and lower extremities, with loss of sensation in the left part of both upper and lower extremities. The patient also complained of suprapubic discomfort and had urinary retention, which raised a suspicion of a possible urinary tract infection. His immunizations were all up to date, and he had no family history of neurological diseases.

During the physical examination, he was febrile (40.0 °C), confused, and oriented only to his name. He had a seizure episode that was relieved with phenytoin. His body mass index was normal (21 kg/m^2^). Ophthalmological examination revealed normal pupillary light reflexes and no obvious cause of vision loss.

## 4. Investigations

**Figure 1:**
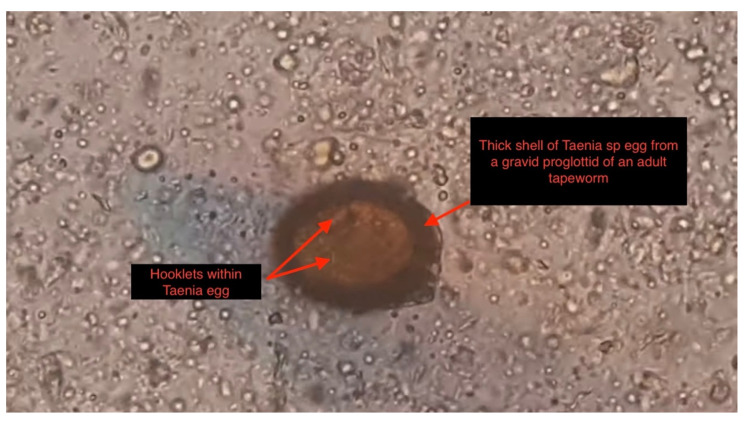
Microscopic slide view (magnified 1600×), Showing a thick wall egg shed from a gravid proglottid of a *Taenia* sp. containing embryo hooklets (red arrows in slide) in the stool sample of our patient three months following symptoms onset.

**Figure 2:**
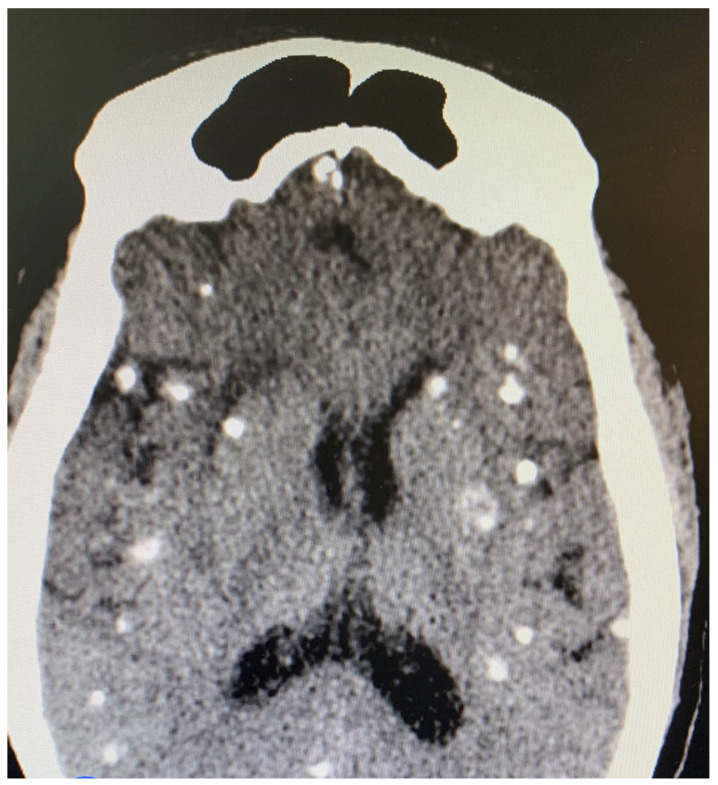
Head CT scan showing multiple cysticerci in the cerebral cortex.

**Figure 3:**
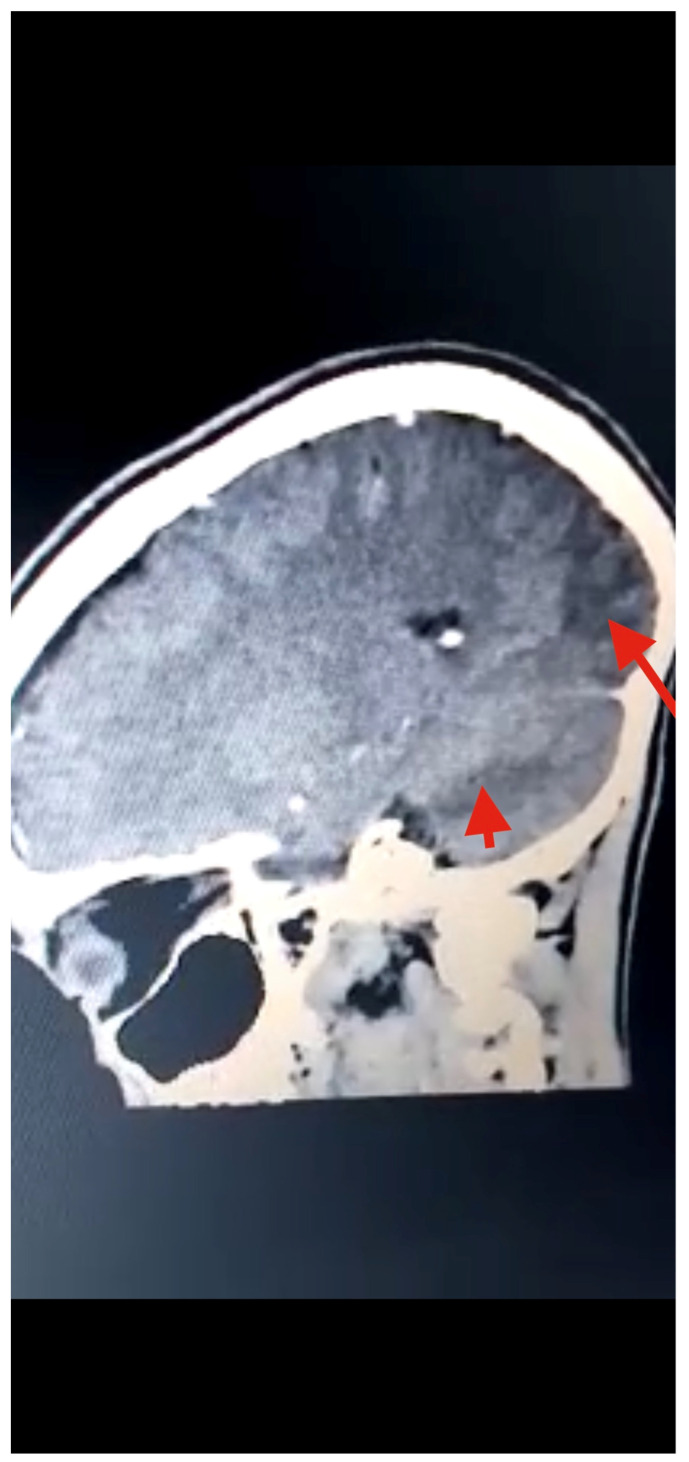
Head CT scan showing multiple T1W hypointense, with T2W and FLAIR hyperintense, areas in the posterior and parietal cortices, indicative of infarction (red arrows) due to stroke from cysticercal invasion of the vascular bed.

Several diagnostic tests were performed, including complete blood count, urinalysis, stool microscopy test Figure 1, and cerebrospinal fluid analysis Table 1. Serology testing (enzyme-linked immuno-electrotransfer blot) of blood for cysticercosis antibodies to glycoprotein antigens was positive, suggesting cysticercosis. Head computed tomography (CT) scan showed local soft tissue inflammation and multiple cysticerci granulomas due to cysticerci degeneration in the cerebral cortex Figure 2. Brain magnetic resonance angiography (MRA) showed a wedge-shaped T1-weighted hypointense and T2-weighted hyperintense lesion in the body of the right caudate nucleus. Diffusion of contrast was restricted on diffusion-weighted imaging. The lesion measured 2.3 cm in diameter and was suggestive of an acute/subacute infarct Figure 3. There was no restriction of contrast diffusion on DW1, suggesting white matter changes. A final diagnosis of cortical blindness due to NCC and co-infection with a urinary tract infection was made.

## 5. Treatment

Management of the patient began with bladder catheterization, which drained 200 mL of cloudy urine and relieved the suprapubic discomfort. Urinalysis showed bacteriuria, and IV levofloxacin 250 mg was administered every 24 h for 3 days. A slow IV infusion of 15 mg/kg phenytoin was also administered to control the seizures. A glucocorticoid (IV methylprednisolone 20 mg every 6 h) was administered to the patient at a lower pressure to reduce inflammation in the brain. An antihelminth drug (oral albendazole 400 mg BID) was also added to the treatment regimen. The patient’s symptoms improved over the course of five days of in-hospital stay. A prescription of oral albendazole at the same dosage was administered to the patient for 10 additional days upon discharge from the hospital.

In addition to the above treatments, visual training was introduced as experimental treatment which consisted of three parts: Restitution therapy aimed at recovering the patient’s visual field deficits during which our patient detected light spots on a dark screen in his bilateral visual fields; Compensation therapy: the patient attempts to capture light stimuli placed on his blind spot; Substitution therapy: which uses specialized devices to project light stimuli from a patient’s blinded peripheral vision to the recovering peripheral visual field. Our patient did not exhibit improvement in his peripheral visual field with the substitution therapy.

## 6. Outcome and Follow-Up

**Figure 4:**
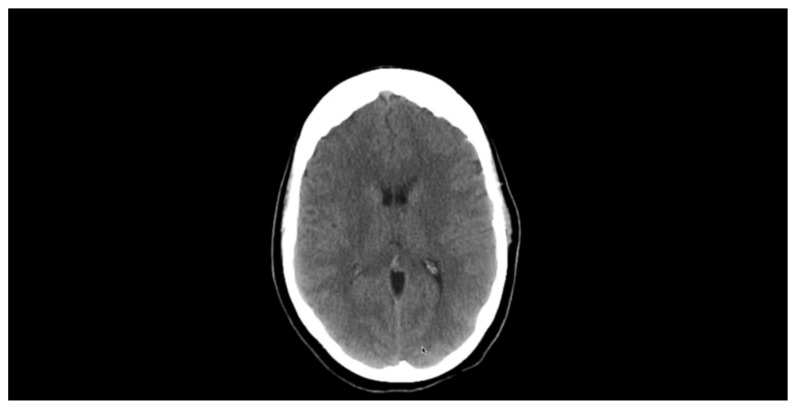
Brain CT showing complete resolution of scolexes following three-month albendazole therapy.

A repeat brain CT with contrast was performed 3 months after discharge from the hospital, and it showed complete resolution of the lesions Figure 4. There was also no further recurrence of the symptoms at the 1-year follow-up visit, and no neurological abnormality was noted during clinical examination of the patient. The patient was able to detect light sources and images following the experimental treatments, but still was unable to describe images that he sees, thus partial bilateral blindness was evident.

## 7. Discussion

NCC is a major cause of morbidity and mortality in people of all ages [5]. Since it is seen as a disease of those with a poor socioeconomic status, the approach towards its control has remained controversial. NCC is known to be the most common cause of adult-acquired epilepsy worldwide, and it causes 30% of the epilepsy cases in most endemic areas of Africa, Asia, and Latin America, particularly in regions where people live in proximity to pigs [6]. Since tourism has become increasingly common in developing countries particularly in Nigeria, comprehensive diagnostic criteria for early detection of NCC have been suggested by Del Brutto et al. [7] which are based on clinical symptoms, neuroimaging, serological, histopathological, and epidemiological approaches. These criteria were used in diagnosing our patient with NCC based on the patient’s positive neuroimaging findings, resolution of the cystic lesions after antihelminth therapy, and travel history to disease endemic areas. Hemiparesis in our patient could be due to extraparenchymal NCC vasculitis caused by inflammatory occlusion of the arteries at the brain stem due to arachnoiditis.

**Figure 5:**
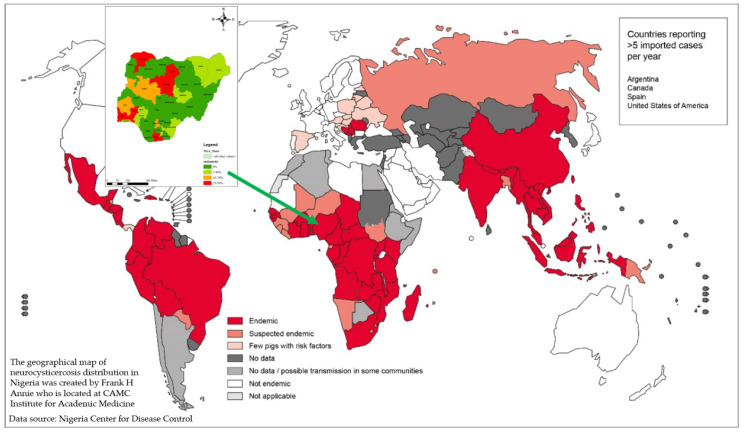
WHO and NCDC *T. Solium* map depicting endemic regions in the world and in Nigeria, respectively [6,12]. Reprinted with permission from ref. [6]. Copyright 2016 World Health Organization and 2018 Nigeria Center for Disease Control.

Visual loss in NCC can be multifactorial. A study showed [8] 23 patients analyzed with vision loss due to NCC. Approximately 50% of the cases were due to optic neuropathy caused by papilledema. The remaining lesions were caused by chiasmal and retrochiasmal lesions [9]. The etiology of cortical blindness due to cysticercosis remains poorly understood. In our patient, we hypothesized that the cause of vision loss was either parenchymal cyst invasion of the arteries of the base of the brain, resulting in vasculitis of the occipital branch of the cerebral artery, or compression of the posterior cerebral artery by large cysts resulting in infarction and subsequent blindness. It is important to note that cysts can spread to every part of the brain, and due to local immune reactions to the presence of these cysts, their stage, location, and size, NCC can present with different neurological symptoms. It is possible that our patient acquired *T. solium* infestation several months prior to symptom manifestation. Several studies have described the unusual clinical manifestations of NCC-like extrapyramidal symptoms, such as hemiballismus [10], dorsal midbrain syndrome [11], and nominal aphasia. Therefore, more research focusing on the newer and more effective treatment strategies for the management of patients with NCC cortical blindness is urgently needed. NCC can be found in any region in Nigeria with poor sanitation, poor veterinary care for especially dogs and pigs, and lack of knowledge on transmission of tapeworms and other helminths in the community Figure 5 [12].

## 8. Conclusions

Neurocysticercosis (NCC) can produce complex neurological symptoms, and in most cases run an indolent course. It is a treatable and a preventable disease. The possibility of this infectious disease should be considered during the diagnostic work-up of any patient with symptoms suggestive of neurological, personality, or cognitive disorders, particularly among patients who have travelled to or lived in an endemic region. NCC damage to the visual cortex in the brain is irreversible, whereas NCC intraocular lesions have shown over 80% improvement with eye surgery. However, in every suspicious case of NCC, prompt diagnosis and treatment are imperative to prevent irreversible neurological damage and death.

## Figures and Tables

**Table 1:** Patient CSF analysis showing moderate pleocytosis with eosinophilia, and slight increase in protein.

Variables	Patient	Normal
Cell count	30 cells (eosinophils)	<5
Glucose	55 mg/dL	50–80 mg/dL
Protein	70 mg/dL	15–60 mg/dL
Opening pressure	100 mmH_2_0	90–180 mmH_2_0

## Data Availability

The original data are included and cited in the article. Further inquiries can be directed to the corresponding author.
